# Exploring Retinopathy of Prematurity: Insights from the First USAID-Funded Screening Program in KPK, Pakistan

**DOI:** 10.12669/pjms.41.7.11021

**Published:** 2025-07

**Authors:** Nazli Gul, Sadia Sethi, Imran Ahmad, Ahmad Mansoor, Zain Ali Nadeem, Wajeha Najeeb, Afshan Hussain Khattak

**Affiliations:** 1 Nazli Gul, FCPS (Ophthalmology, Pediatric Ophthalmology& Strabismus), CHPE Professor & Chairperson, Department of Ophthalmology, MTI-Khyber Teaching Hospital, Peshawar, Pakistan; 2 Sadia Sethi, FCPS (Ophthalmology), CHPE Professor, Department of Ophthalmology, MTI-Khyber Teaching Hospital, Peshawar, Pakistan; 3 Imran Ahmed, FCPS (Ophthalmology, Vitreo-Retina), CHPE, MHPE Associate Professor, Department of Ophthalmology, MTI-Khyber Teaching Hospital, Peshawar, Pakistan; 4 Ahmad Mansoor, MBBS-IV Services Institute of Medical Sciences, Lahore, Pakistan; 5 Zain Ali Nadeem, MBBS-IV Demonstrator, Department of Community Medicine, Allama Iqbal Medical College, Lahore, Pakistan; 6 Wajeha Najeeb, MBBS CMH Kharian Medical College, Kharian Cantt, Pakistan; 7 Afshan Hussain Khattak, MD, FAAP Director, SPIRE, Pakistan

**Keywords:** Diagnostic screening programs, Retinopathy of prematurity, Southern Asia, Pakistan, USAID

## Abstract

**Objective:**

To investigate incidence, demographics and risk factor associated with Retinopathy of prematurity.

**Methods:**

It was multi-centered prospective cross sectional study. Department of Ophthalmology at Khyber Teaching Hospital conducted this study, from February 2022 to September 2023 in collaboration with three teaching hospitals based in Peshawar. It was funded by United States Agency for International Development (USAID) with Grant No. CBP051. Infants born at ≤ 35 weeks, or birth weight ≤ 2kg, or admitted to Neonatal Intensive Care Unit (NICU) for ≥ three days were included. Screening was conducted at ≥3-4 weeks after birth or 31 completed weeks post-conception, whichever came first. Endpoint for follow-up was complete vascularization of temporal retina.

**Results:**

It included 622 of 840 screened infants, with 98(15.8%) ROP cases. Among diagnosed, 56.1% were males, 66.3% born between 26-30 weeks, and 63.3% with birth weight between 0.6-1.5 kg. 62.2% were at 1-4 months of age, 98.9% had screening weight range 1-3 kg, 99% received postnatal oxygen therapy, and 24.5% had neonatal diseases. In multivariate analysis, risk factors including gestational age (AOR 0.793, 95% CI 0.69-0.90), postnatal oxygen therapy (AOR 40, 95% CI 1.9-817), duration of postnatal oxygen therapy (AOR 1.05, 95% CI 1.02-1.07), birth weight (AOR 0.3, 95% CI 0.125-0.725), blood transfusion (AOR 349, 95% CI 17.7-9601), and neonatal diseases (AOR 2.4, 95% CI 1.2-4.8) were found significant.

**Conclusions:**

Gestational age, postnatal oxygen therapy and neonatal disease are significant factors associated with ROP.

## INTRODUCTION

Retinopathy of prematurity (ROP), is characterized by excessive proliferation of retinal blood vessels in preterm infants, which impair vision permanently if left untreated. Global preterm birth rate is approximately 11%, with about 15 million births annually.^1^ Preterm infants in neonatal intensive care units (NICU) often require oxygen therapy, which can trigger oxidative stress and inflammation. This in turn dysregulate signaling pathways, leading to reduction in vascular endothelial growth factors (VEGF) and halting retinal vascularization. Discontinuing oxygen therapy subsequently upregulate VEGF, causing retinal neovascularization which grow in an uncontrolled and disorganized manner, leading to ROP.^2^

ROP follows a predictable course, and early detection is crucial. Several risk factors have been associated with ROP, including low birth weight, low gestational age, late onset sepsis, and oxygen therapy (mechanical ventilation, noninvasive ventilation).^3^ American Academy of Pediatrics recommends timely retinal examination for infants with birth weight <1.5kg or born <30 weeks of gestation.^4^ With advancements in prenatal care, survival rates of low-birth-weight preterm infants are increasing, especially in developed countries. In low and middle-income countries, incidence is higher due to factors including preterm deliveries, lack of awareness, financial challenges, and insufficient treatment and screening facilities.^5^ Blindness and visual impairment caused by ROP are significant causes of preventable blindness and economic burden, particularly in Asia and Sub-Saharan Africa.^6^

Despite being a resource-poor country, Pakistan has witnessed a steady improvement in survival of premature newborns. Unfortunately, Pakistan lack sufficient follow-up programs for these children, and even more scarce is screening and treatment of special healthcare needs, such as ROP. Some ROP screening programs previously were conducted in Islamabad^7^ or Karachi,^8^ none were done in Khyber Pakhtunkhwa (KPK). This highlight the need to conduct studies in different regions of Pakistan to obtain a more representative prevalence of ROP and to devise a screening protocol for our population. In this study, we present the results of the first-ever United States Agency for International Development (USAID) funded ROP screening program conducted by Department of Ophthalmology at Khyber Teaching Hospital in Peshawar.We also aimed to establish a comprehensive database of families with premature infants with Retinopathy of Prematurity (ROP) to identify the demographic characteristics and clinical course in Neonatal Intensive Care Unit (NICU).

## METHODS

A multi-centered prospective cross-sectioned study was envisioned through collaboration of consultant neonatologist, pediatricians and ophthalmologists from three teaching hospitals based in Peshawar Khyber Teaching Hospital, Hayatabad Medical Complex, and Town Children’s Hospital. The Department of Ophthalmology at Khyber Teaching Hospital (KTH) conducted the study, which was funded by USAID with Grant No. CBP051. Ethical approval was granted by Khyber Medical College’s Institutional Research Board 8/8/DME/KMC, dated: February 22, 2023.

The duration of study was from February 2022 to September 2023. Social Action for Progress, Innovation, Research and Education (SPIRE) was consulted to assist in maintaining and analyzing the extensive database kept in KTH. SPIRE worked under Memorandum of Understanding (MOU), SPIRE VISION 03/2023 with department of ophthalmology at KTH. Arrangements were made for developing and securing of this Excel based database in Nursery/NICU of KTH and SPIRE’s Peshawar office. The study focused on the population of Peshawar. There are seven registered NICUs in tertiary care centers with ophthalmology services, only two had trained staff capable of conducting ROP screening and follow-up services, with KTH being one of them. There are approximately ten Level-1 and two level-2 NICUs within the city and four rural Level-1 NICUs outside Peshawar. The SPIRE-ROP team reached out to all units with no onsite ROP screening services for collaboration and participation. They were instructed to refer the patients fulfilling inclusion criteria to the study site, convincing them to take part in the initiative taken to provide better neonatal health services.

Infants born at ≤ 35 weeks of gestation with birth weight of ≤ 2 kg and, remain admitted in NICUs ≥ three days were enrolled. An eye examination for ROP screening was performed at 3–4 weeks after birth or 31 completed weeks post-conception depending upon whichever came first. Infant’s pupils were dilated with 1% tropicamide and 10% phenylephrine diluted with artificial tears in 1:4. One drop was instilled in each eye five minutes apart for three times. In case of inadequate dilation, an additional eye drop was instilled after 30 minutes. The consultant ophthalmologist performed a detailed retinal examination with an indirect ophthalmoscope, infant speculum, and scleral indentation. ROP was graded into stages and zones based on the International Classification of Retinopathy of Prematurity.^9^ The endpoint for our ROP follow-up was complete vascularization of temporal retina.

Informed consent was obtained from the parent/guardian before ROP screening. The core ROP screening team comprised of a consultant ophthalmologist, two trainee ophthalmologists, one neonatologist, one paediatrician with special interest in neonatal care, and two paramedical staff members. The study team was equipped with portable 20D and 28D indirect keeler specialist ophthalmoscope. NICU staff was trained to inform the ROP coordinator about infants meeting the inclusion criteria. Parents or guardians of enrolled premature infants were counselled regarding the critical importance of ROP screening and the need for further management to prevent blindness if ROP was detected. A consultant ophthalmologist, based in KTH, Peshawar, performed ROP examinations on a list of referred infants thrice a week.

Infants who required treatment for ROP were administered intravitreal Anti VEGF injections (Anti Vascular Endothelial Growth Factor). Laser treatment was conducted on-site if indicated, using the Iridex Laser from the USA. Infants who required vitreoretinal surgery were referred to specialized ophthalmology services in Lahore, the capital city of Punjab, Pakistan, for further intervention.

Data was collected from 840 enrolled infants, but after screening and cleaning for errors and participant dropouts, 622 were included in the final analysis. Key variables—gestational age, birth weight, weight and age at screening, and duration of postnatal oxygen therapy—were analyzed as continuous variables, with means and standard deviations reported. These variables were also categorized for further analysis. Categorical variables included postnatal oxygen therapy, gender, neonatal diseases, and blood transfusion during the neonatal period. In univariate analysis, we calculated frequencies and percentages for demographic characteristics, focusing on patients diagnosed with ROP. We used Pearson chi-square, Fisher’s exact test, or likelihood ratios for categorical variables related to ROP. For multivariate analysis, binary logistic regression identified risk factors for ROP. The model demonstrated a significant fit, with an omnibus test p-value of 0.001 and a good fit according to the Hosmer and Lemeshow test (non-significant p-value). The variance explained by the model, as indicated by Nagelkerke R square, ranged from 24% to 42.2%. The model achieved 87% accuracy, 97.5% specificity, and 30.6% sensitivity.

## RESULTS

Of 840 infants screened, 622 records were included in final analysis. 387(62.2%) were male and 235(37.8%) were female infants. Mean gestational age, neonatal age at screening, weight at birth and at screening is described in Table-I. In terms of gestation outcomes, a significant majority of the cases, 442 (71.1%), involved single pregnancies, while 136 (21.9%) were multiple pregnancies. Additionally, 44 cases (7.1%) represented multiple pregnancies in which one or two siblings had died perinatally. Among all, 509 (81.8%) had received postnatal oxygen therapy. Maximum duration of postnatal oxygen therapy received by any of the participants was 90 days, with mean and standard deviation of 8.46±9.56 days. History of blood transfusion was found in 17(2.7%) neonates and 96(15.4%) had associated disease at birth.

**Table-I:** Demographic characteristics of study population.

Sr.	Variables	Unit of measurement	Minimum value	Maximum value	Mean±SD
1	Gestational age	Weeks	24	38	31.13±2.47
2	Weight at birth	Kilograms	0.50	3.10	1.65±0.37
3	Infant/neonatal age at screening	Days	3	330	45.73±37.64
4	Infant/neonatal weight at screening	Kilograms	0.90	6.70	2.27±0.79

Respiratory distress syndrome (RDS) being the most common neonatal disease, affecting 46(7.4%) neonates, followed by neonatal jaundice (NNJ), affecting 16(2.7%), 12(1.9%) had sepsis, 7(1.1%) had both RDS and NNJ, 4(0.6%) had RDS and sepsis, and 3(0.5%) had NNJ and fits. Two infants (0.3%) had lower respiratory tract infections (LRTI), and two had LRTI with sepsis. One infant had sepsis with congenital heart disease, one had NNJ with sepsis, and one had both LRTI and RDS. One infant had thick meconium-stained liquor at birth. Ocular disease at birth were diagnosed in 24(3.9%) participants, which included persistent hyperplastic primary vitreous, uniocular retinoblastoma, microspherophakia, and uveal coloboma. Among all infants 98 (15.8%) were diagnosed with ROP.

Among those diagnosed with ROP, greater proportion were males, born between 26-30 weeks of gestation, had weight ranging from 0.6 to 1.5kg, were at 1-4 months of age at the time of screening and had neonatal weight of 1-3kg. (Table-II) Almost all diagnosed cases received postnatal oxygen therapy, had some neonatal disease. Additionally, 94.1% who received transfusion were affected with ROP. In univariate analysis, gestational age and weight at birth, postnatal oxygen therapy, blood transfusion, and neonatal disease were seen to be significant risk factor for ROP (p-value ≤0.05) (Table-II).

**Table-II:** Univariate analysis of risk factors for Retinopathy of prematurity (ROP).

Sr.	Variables	Subcategories	ROP Affected	ROP Unaffected	p-value
1	Gender	Male	55(56.1%)	332(63.4%)	0.18^a^
		Female	43(43.9%)	192(36.6%)	
2	Gestation	Single	67(68.4%)	375(71.6%)	0.648^a^
		Multiple	22(22.4%)	114(21.8%)	
		Multiple with 1-2 siblings expired	9(9.2%)	35(6.7%)	
3	Gestational age	≤25 weeks	1(1%)	3(0.6%)	0.001*^c^
		26-30 weeks	65(66.3%)	181(34.6%)	
		31-35 weeks	32(32.7%)	335(64.1%)	
		≥36	0	4(0.8%)	
4	Weight at birth	≤0.5 kg	0	1(0.2%)	0.001*^c^
		0.6-1.5 kg	62(63.3%)	179(34.2%)	
		1.6-2.5 kg	36(36.7%)	341(65.1%)	
		≥2.6kg	0	3(0.6%)	
5	Infant/neonatal age at screening	< 1 month	34(34.7%)	236(45%)	0.165^c^
		1-4 months	61(62.2%)	265(50.6%)	
		5-8 months	3(3.1%)	21(4%)	
		9-12 months	0	2(0.4%)	
6	Infant/neonatal weight at screening	< 1kg	0	1(0.2%)	0.817^c^
		1-3 kg	90(98.9%)	464(98.9%)	
		> 3kg	1(1.1%)	4(0.9%)	
7	Postnatal oxygen therapy	Yes	97(99%)	412(78.6%)	0.001*^a^
		No	1(1%)	112(21.4%)	
8	Blood transfusion (postnatal)	Yes	16(16.3%)	1(0.2%)	0.001*^b^
		No	523(99.8%)	82(83.7%)	
9	Neonatal disease	Yes	24(24.5%)	72(13.7%)	0.009*^a^
		No	74(75.5%)	452(86.3%)	

* p-value ≤ 0.05,

a p-value calculated via Pearson chi square,

b p-value calculated via Fisher exact test,

c p-value calculated via Likelihood ratio.

**Fig.1:**
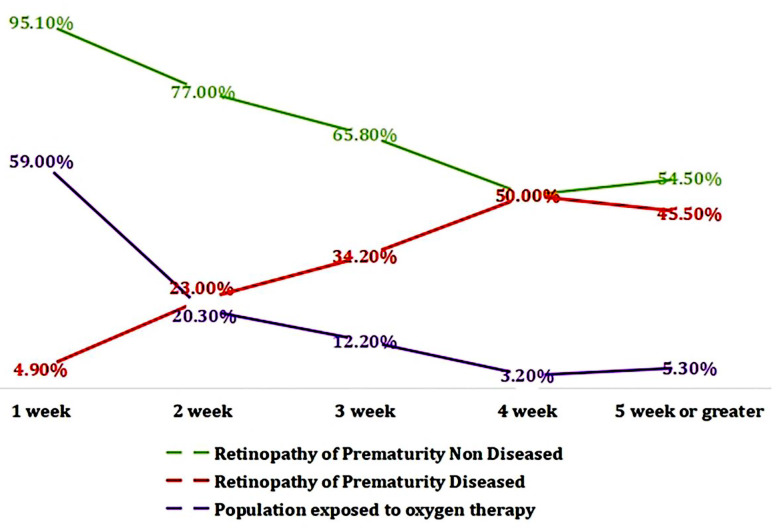
Relationship between duration of postnatal oxygen therapy and frequency of ROP.

A crucial connection was found between postnatal oxygen therapy and the prevalence of ROP, (likelihood ratio, p-value <0.001) as illustrated in Fig.1. A significant increase occurred in number of neonates diagnosed with ROP as duration of exposure to postnatal oxygen therapy increased in weeks (Fig.1). Binomial logistic regression was applied to evaluate impact of demographic variables on presence or absence of ROP as described in Table-III. Analysis revealed that birth, gender, and ocular disorders were not found to be associated with ROP (Table-III). However, it was found that for every unit increase in gestational age, there was a decrease in the likelihood of developing ROP. Additionally, the analysis revealed that patients who were given postnatal oxygen had 40 times higher risk of developing ROP. It was also observed that for every additional day of postnatal oxygen received, there was a one-time increase in the risk of developing ROP.

**Table-III:** Multivariate (Binomial logistic regression) analysis of risk factors for Retinopathy of prematurity (ROP).

Sr.	Variables	Units of measurements	OR	95% Confidence Interval		p-value
				Lower	Upper	
1	Gestational Age	Weeks	0.793	0.698	0.902	0.001*
2	Gender	Female (reference)/Male	0.755	0.443	1.290	0.304
3	Weight at birth	kilograms	0.301	0.125	0.725	0.007*
4	Infant/neonatal age at screening	Days	0.987	0.977	0.998	0.017*
5	Infant/neonatal weight at screening	Kilograms	0.964	0.568	1.635	0.892
6	Gestation	Single	reference	reference	reference	0.830
		multiple	0.889	0.455	1.737	0.730
		Multiple 1-2 expired	0.757	0.280	2.047	0.583
7	Blood transfusion (postnatal)	No(reference)/yes	349.5	17.708	6901.6	0.001*
8	Neonatal diseases	No(reference)yes	2.416	1.201	4.860	0.013*
9	Ocular disorder	No(reference)/ yes	0.908	0.117	7.053	0.927
10	Postnatal oxygen therapy	No(reference)/ yes	40.183	1.976	817.054	0.016*
11	Duration of Postnatal oxygen therapy	Days	1.050	1.021	1.079	0.001*

* significant p-value.

Similarly, an increase in the weight of neonates at birth was associated with a lower risk of ROP. Moreover, Patients who received postnatal blood transfusions were found to have a 349 times higher risk of developing ROP. Lastly, the risk of ROP among patients with any associated neonatal disease was also found to be two times greater than those without any associated neonatal disease.

## DISCUSSION

This study found that 15.8% of neonates had ROP, which aligns with data from other local studies where ROP incidence is around 15.9%.^10^ Reported incidence of ROP varied across different regions of Pakistan, ranging from 3.2% to 28%.^7^-^9^ Notably, in Iran it was around 33.9%^11^, while in developed countries it varied from 16.2% to 39.6%^12^-^15^ The observed differences in incidence may result from the retrospective nature of studies, smaller population sizes, screening age limits, inclusion criteria, and variations in neonatal care. By broadening our inclusion criteria, helped in timely diagnosis of more patients.^16^ Moreover, genetic variations^17^ were also seen to be associated with ROP, indicating complex and multifactorial nature of disease.

Male preterm infants had higher incidence of ROP (56.1%). Similar findings were reported by Yu CW et al., with 52.3% of males affected.^18^ Lin et al., also found male gender as one of the risk factors for ROP.^19^ Additionally, our study identified low gestational age and lower birth weight as two of most common risk factors, which is supported by many other studies.^15^,^20^ Contrary to that, Daneshtalab A et al.^21^, reported that gestational age is not a significant risk factor in multivariate analysis whereas, Junling MA et al., specifically indicated that lower gestational age is strongly associated with a higher incidence.^22^ Infants weighing less than 1.5kg had higher ROP incidence of 63.3% compared to those weighing 1.6-2.5kg i.e. 36.7%. These results align with study by Fortes Filho JB et al. which concluded that infants weighing less than one thousand grams had severe ROP.^23^ In developing countries, lower socio-economic status may lead to lower compliance with ROP screening appointments among patients wih lower birth and gestational age, which could contribute to their vulnerability to this condition.^24^

In postnatal oxygen therapy, there is 40 times higher risk of developing ROP. This risk increases with each additional day of oxygen therapy. Infants requiring oxygen for four weeks had highest incidence of ROP at 50%, followed by 45.5% for five weeks or more, and 34.2% for three weeks. Similar results were found by Yadav et al.^20^ and Di Pietro M et al.^25^ showing that the duration of assisted ventilation was significantly associated with ROP development (p-value <0.001). Mechanical ventilation is also identified as a risk factor.^15^ In contrast, a Malaysian retrospective cohort study including data from 44 NICUs found that receiving oxygen therapy at birth was associated with a significantly lower risk of ROP development.^26^ It is essential to note that our study did not observe the fraction of inspired oxygen (FiO2) or the method of oxygen delivery, whether through mechanical or non-invasive ventilation. We only monitored the oxygen supplementation and the number of days of oxygen therapy. Additionally, patients who received postnatal blood transfusions showed 349 times higher risk of ROP development, several studies further supported this association.^15^,^20^ The risk was also twice as high in patients with any postnatal disease as compared to those without any neonatal disease. The neonatal diseases observed were RDS, NNJ, sepsis, fits, LRTI, and CHD, which were also noted as risk factors in other studies.^20^,^26^

However, it is important to note that this study did not take into account several neonatal diseases and the individual effect of each disease on ROP. It is crucial to acknowledge that various risk factors for ROP are preventable, such as monitoring postnatal oxygen, age and weight at screening, gestational age, and weight at birth by provision of good prenatal and neonatal care. Non-modifiable risk factors include neonatal diseases at birth, including ocular diseases, blood transfusions, and gender. Thus, concluded that by implementing primary prevention strategies we can modify risk factors.^4^

Our study employed a multicenter approach and included a substantial sample size, focusing on a screening program designed to identify risk factors associated with retinopathy of prematurity (ROP) and determine its prevalence among neonates in the KPK region. We identified key high-risk factors, including gestational age at birth, birth weight, use of postnatal oxygen therapy, postoperative blood transfusions, and the presence of neonatal diseases. Addressing and managing these factors is essential to reduce the incidence of ROP. This study suggested that the current criteria, which focus on infants born at <32 weeks of gestation and weighing <1.5 kg, may be inadequate for effective enrollment. It is vital to incorporate a broader range of risk factors into ROP management to better understand the disease’s pathophysiology and its associated comorbidities. Further research is needed to comprehensively analyze maternal factors, mode of delivery, and neonatal characteristics, as well as to monitor parameters such as oxygen saturation, variations in oxygenation, postnatal growth, and nutrition, all of which may impact ROP. Our study primarily addressed only a limited number of risk factors and associated neonatal diseases, highlighting the need for a more thorough examination in future research, along with reevaluating the current screening processes and revising existing guidelines.

### Limitations:

Our research has identified a few limitations. The significant one is in determining complications associated with ROP in preterm infants due to a lack of follow-up.

## CONCLUSIONS

This screening program identified gestational age, postnatal oxygen therapy, duration of postnatal oxygen therapy, birth weight, screening age, blood transfusion, and neonatal diseases as the significant risk factors of ROP. Understanding and addressing these risk factors could potentially help in the prevention and management of ROP in preterm infants and to reduce the burden of this disease in vulnerable populations.
